# Role of Food Safety Risk Perception and Attitude on Knowledge and Practice of Adult Consumers in Bangladesh: A Serial Mediation Model

**DOI:** 10.1002/fsn3.71540

**Published:** 2026-02-12

**Authors:** Rakia Ishra, Saif Sharif, Jeffrey Soar, Rasheda Khanam

**Affiliations:** ^1^ Department of Public Health American International University ‐ Bangladesh Dhaka Bangladesh; ^2^ University of Southern Queensland Toowoomba Queensland Australia

**Keywords:** attitude, consumer, food safety, KAP, knowledge, risk perception, serial mediation

## Abstract

This study aimed to determine the serial mediation effects of behavior‐specific risk perception (BRP) and attitude (ATT) toward food safety on the relationship between food safety knowledge (FSK) and self‐reported practice (FSP) among adult consumers in Bangladesh. This study conducted a cross‐sectional survey on cross‐contamination, safe storage, and cooking toward food safety among 503 consumers who handled food at home. The serial mediation effects were examined by using Hayes' PROCESS macro‐Model 6 for SPSS. The results showed that BRP partially mediated the relationship between FSK and FSP independently (Effect = 0.175, 95% CI: 0.114 to 0.243). BRP was significantly positively associated with ATT (Effect = 1.28, *p* < 0.001) and FSP (Effect = 0.532, *p* < 0.001), forming a serial mediation pathway (Effect = 0.065, 95% CI: 0.037 to 0.099). The total indirect effect of all mediation paths accounted for 50% of the total effect of food safety knowledge on food safety practice.

## Graphical abstract

Using cross‐sectional data from 503 Bangladeshi adult consumers and applying Hayes' PROCESS macro for serial mediation analysis, this study shows that food safety knowledge affects food safety practices both directly and indirectly through behavior risk perception and attitude. Risk perception partially mediated the knowledge–practice relationship and fostered positive attitudes, forming a significant serial mediation pathway. Overall, indirect effects accounted for nearly half of the total effect, highlighting the critical role of psychosocial factors in improving domestic food safety practices.

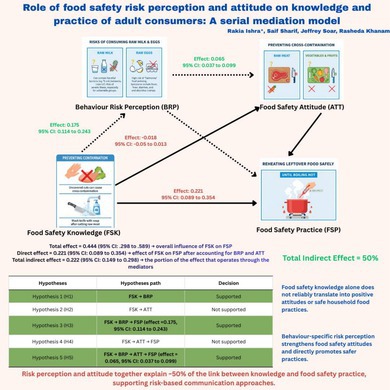

## 1 Introduction

Foodborne diseases (FBDs) have impacted the global and domestic food sectors, posing a significant hazard to public health. The World Health Organization (WHO) estimates that unsafe food is responsible for illness in almost 1 in 10 individuals globally, approximately 600 million individuals. As a result, there are 420,000 fatalities and a loss of 33 million healthy life years (DALYs) each year (WHO 2022). The burden of FBDs is much worse in developing nations (Lee and Lien 2015; Wang et al. 2021). Owing to its dense population, inadequate infrastructure, limited access to clean water, and absence of sanitation and hygiene (WASH) facilities, FBDs are prevalent in low‐ and middle‐income countries such as Bangladesh (Ishra et al. 2022). Approximately 30 million individuals in Bangladesh suffer from FBDs annually (Ishra et al. 2023).

Earlier research has reported that handling food at home accounted for over 35% of FBDs (Langiano et al. 2012). The prevalence rate of FBD detected in the household kitchen greatly varied within nations (Al‐Shabib et al. 2016; Islam et al. 2023). Consumers who handle food at home are a significant cause of food contamination because hazardous pathogens can be transmitted through cross‐contamination of food handlers' hands (Baş et al. 2006; Campos et al. 2009; Kunadu et al. 2016). Cross‐contamination accounts for most FBD outbreaks in households (Redmond and Griffith 2003); for example, without routine washing, using a similar cutting board and knife set for poultry, raw meat, and vegetables can lead to FBD (Almanasrah et al. 2022; De Jonge et al. 2010). In an earlier study, over 60% of consumers in Bangladesh failed to demonstrate the correct way of cross‐contamination practices. For instance, using a chopping board for raw meat and fresh fruit was shown to have the lowest level of comprehension (19.9%). Safe storage is another key procedure to control foodborne pathogens. Around 75% of Bangladeshi consumers failed to indicate safe food storage, such as handling freshly cooked food that would not be eaten for three or more hours (Islam et al. 2023). One efficient strategy to lower FBDs is to improve the food‐handling practices of consumers. Therefore, consumers should have an appropriate food safety knowledge level to understand and practice safe handling, preparation, and storage of food (Young et al. 2017).

Numerous studies have been conducted to evaluate consumer food safety behavior throughout the world through the KAP (knowledge, attitude, and practice) model (Agüeria et al. 2018; Farahat et al. 2015; Kunadu et al. 2016; Luo et al. 2019; Moreb et al. 2017; Sanlier 2009; Tomaszewska et al. 2018). The KAP theory emphasizes the value of knowledge and attitude in supporting practices (Mihalache et al. 2021; Zanin et al. 2017). This model only includes two variables, knowledge and attitudes, and explains a complex phenomenon. However, this linear construction may oversimplify the complex food safety behavior. The correlation between KAP has shown variability in different research studies and contexts (da Cunha et al. 2022). For example, some research suggests that food safety knowledge and practice are weakly correlated, and that increasing food safety knowledge does not always lead to healthier food safety practices (Gong et al. 2016; Kwol et al. 2020).

Behavior theories such as the Protection Motivation Theory (PMT) and Health Belief Model (HBM) may provide an effective theoretical basis to address these limitations (Young et al. 2017). These frameworks emphasize the importance of risk perception, enabling individuals to make rational decisions that lead to appropriate food‐handling behaviors when they believe that food‐related risks may be potentially serious and personally relevant (Young et al. 2017). Therefore, incorporating risk perception into the KAP framework provides a theoretically grounded approach to explain why attitudes and knowledge do or do not translate into safe practices. Although risk perception is an important phenomenon, there is limited evidence of how risk perception affects the KAP factors. This study investigates consumers' cross‐contamination, safe storage, and cooking behavior by applying the KAP model, including risk perception through a serial mediation model.

## 2 Theory and Hypothesis

### 2.1 The KAP Theory

**FIGURE 1:**
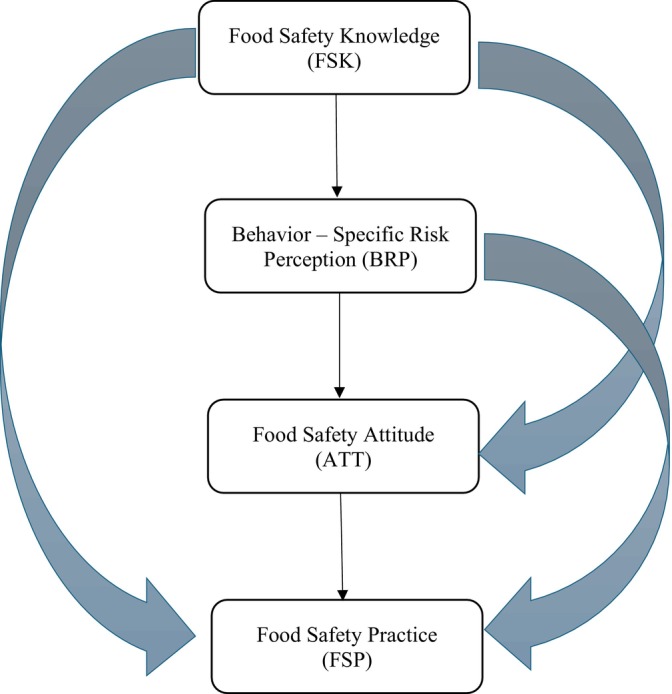
Proposed conceptual model based on the extended KAP framework showing hypothesized directional effects among the study constructs.

Models for behavioral transformation commonly include knowledge, attitudes, and practices (Liao et al. 2022). The utilization of the knowledge, attitude, and practice (KAP) model served as the theoretical basis for formulating the hypothesized associations in this study. According to the KAP model, knowledge positively impacts attitudes, and attitudes influence practices. However, empirical evidence suggests that the strength and direction of these relationships are not universally consistent and are context dependent. In the realm of food safety, knowledge plays a vital role in shaping consumer attitudes and subsequent behaviors concerning cross‐contamination, safe storage, and cooking (Zanin et al. 2017; Ishra et al. 2025b). Over the years, several studies have sought to apply the model to examine the attitudes and actions of food handlers in various circumstances, with diverse findings regarding the translation of knowledge into attitudes and practices (Agüeria et al. 2018; Farahat et al. 2015; Kunadu et al. 2016; Luo et al. 2019; Moreb et al. 2017; Sanlier 2009; Tomaszewska et al. 2018). In light of these inconsistencies, this study examines behavior‐specific food safety risk perception in the classic KAP model to better explain how food safety knowledge translates into domestic food‐handling practices (Figure 1).

### 2.2 Consumer Food Safety Knowledge and Behavior Risk Perception

Consumer food safety knowledge significantly influences their level of food safety practices (Gong et al. 2016). While it is necessary to possess food safety knowledge to implement good practices, research has shown that this knowledge alone is not adequate to perceive food risks (Redmond and Griffith 2003; Fischer et al. 2007; Bearth et al. 2014). As a case in point, prior research has documented that despite most individuals having adequate knowledge about the crucial role of cooking in preventing FBDs caused by cross‐contamination, it did not necessarily manifest in their practice (Fischler et al. 2007; Phang and Bruhn 2011). Additionally, the preceding research indicates that motivation has a significantly greater influence on consumer food safety practices than food safety knowledge (Fischer et al. 2007; Taché and Carpentier 2014). A previous study found that risk perception is a commonly observed motivating factor in food handling, becoming a reliable predictor of food safety behaviors (Young et al. 2017).

An individual's evaluation of the potential risk in a particular scenario is known as their perception of risk (Slovic 1987; Zanetta et al. 2022; Ishra et al. 2025a). Several behavior theories, such as the Health Belief Model (HBM) and Protection Motivation Theory (PMT), also suggest that increased perception of risks inclines consumers to adopt normative food‐handling behaviors (Young et al. 2017). Furthermore, knowledge often shapes the individual's perception of risk and validates the ability to accurately appraise one's risk of an occurrence and its repercussions (Ning et al. 2020). The earlier study mentions that consumer behavior‐specific risk perception showed a more constructive instrument for altering food handling behavior (Young et al. 2015). Hence, consumers' cross‐contamination, safe storage, and cooking‐related food safety knowledge (FSK) will form their behavior risk perception (BRP) and, consequently, safe food handling practices (Figure 1). Therefore, this study hypothesized:

Hypothesis 1 *Consumer cross‐contamination, storage, and cooking‐related food safety knowledge has a positive effect on behavior‐specific risk perception* (*BRP*) *in the domestic environment*.

### 2.3 Consumer Behavior Risk Perception and Attitude Toward Food Safety

Attitude is frequently conceptualized as a motivating factor that transfers knowledge into practice (Zanin et al. 2017). Attitude is a preexisting tendency to respond to an object rather than actual conduct toward that object. An individual's attitude is considered a permanent trait that can be altered through prolonged periods of pressure and time. This dormant variable has significant behavioral consequences and is primarily associated with a predisposition toward evaluations or feelings toward an object. Despite being comparatively stable psychological orientations, attitudes are not fixed and can change over time in response to contextual constraints, societal factors, and long‐term experiences (Niewczas‐Dobrowolska 2022). Hence, attitude is a latent cognitive‐affective construct that has the potential to impact behavioral decision‐making significantly.

The KAP model suggests that knowledge shapes an individual's attitude, which, in turn, influences their practices. Numerous studies have discussed this proposition and analyzed attitude as a motivating factor (Baser et al. 2017; Sani and Siow 2014; Sanlier and Baser 2020; Taha et al. 2020). However, empirical data indicate that this knowledge–attitude relationship is not always consistent, for example, in routine or habitual behavior such as handling food at home. Food safety procedures are frequently ingrained in established routines in domestic environments and are impacted by cultural norms, past experiences, and perceived vulnerability to foodborne hazards. As a result, knowledge about food safety may continue to be primarily cognitive and may not be sufficiently prominent to cause a major shift in attitudes. Contextual cues, past experiences, social norms, and perceived risks may therefore influence attitudes toward food safety in addition to knowledge (Akabanda et al. 2017; Rebouças et al. 2017). Based on the KAP model, and acknowledging these moderating influences, consumers with adequate knowledge on cross‐contamination, safe storage, and cooking‐related issues will construct their appropriate attitude level (Figure 1). Thus, this study hypothesized:

Hypothesis 2 *Consumer cross‐contamination, storage, and cooking‐related food safety knowledge (FSK) has a direct effect on food safety attitude (ATT) in the domestic environment*.

### 2.4 Mediating Role of Behavior Risk Perception and Attitude

The notion of risk pertains to the likelihood of encountering hazards. Hazards are characterized as dangers that endanger the safety of individuals and their possessions. Probability is related to the possibility of a hazard happening, perceived with some uncertainty (Slovic 2016). Importantly, even when exposed to similar hazardous information, people's interpretations and assessments of risks differ greatly, leading to varied risk perceptions (Slovic 1987). Such subjective risk perceptions are crucial in food safety contexts because they shape awareness of microbiological hazards and direct food‐handling practices, such as hand sanitation, cross‐contamination prevention, and appropriate storage and cooking techniques (De Boer et al. 2005; McCarthy et al. 2007).

Risk perception has been widely conceptualized as a cognitively and affectively salient antecedent to evaluative judgments and attitudinal responses. Studies suggest people who are more aware of the risks associated with food are more concerned, vigilant, and motivated to take precautions, resulting in more positive views about food safety procedures (Slovic et al. 2004; van der Vossen‐Wijmenga et al. 2022). In domestic food‐handling environments, where behaviors are often habitual and shaped by prior experience, risk perception may serve as an activation mechanism that brings otherwise latent knowledge into conscious evaluation, subsequently influencing attitudinal orientations.

However, other theoretical stances recognize that there may be a reciprocal relationship between attitude and risk perception. Preexisting attitudes can affect how risks are perceived and weighed. For example, individuals with positive attitudes toward certain food safety practices may minimize the risks involved, whereas those with negative attitudes may increase risk perceptions (Siegrist 2000). This perspective aligns with theories of cognitive consistency, which contend that individuals search for consistency in their attitudes, beliefs, and assessments of risk, occasionally modifying perceived risks to conform to preexisting evaluative orientations (Festinger 1957; Ajzen 1991).

Despite this potential bidirectionality, this study used a sequential ordering in which behavior‐specific risk perception precedes attitude formation. This decision is grounded in the assumption that food safety knowledge first raises awareness of specific risks and their consequences, such as risks related to cross‐contamination, improper storage, and unsafe cooking, hence elevating perceived behavior risks. This heightened risk awareness is then expected to inform broader evaluative attitudes toward food safety practices. Such cognitive‐to‐evaluative progression is particularly relevant for routine domestic food‐handling practices, where risk salience may be required to interrupt ingrained habits and encourage attitudinal change (De Boer et al. 2005; McCarthy et al. 2007).

Building on this rationale, it is anticipated that food safety knowledge positively influences consumers' perception of behavioral risks related to domestic food handling (Kwol et al. 2020). Consistent with empirical evidence identifying risk perception as an antecedent to attitude development in food safety contexts, in turn, elevated behavior‐specific risk perception is then thought to promote more positive attitudes toward food safety (van der Vossen‐Wijmenga et al. 2022). Together, these mechanisms suggest a sequential mediation pathway whereby behavior‐specific risk perception and attitude jointly transmit the effect of food safety knowledge to food safety practices (Figure 1). Accordingly, the following hypotheses are given:

Hypothesis 3 *Consumer BRP mediates the relationship between FSK and FSP levels in the domestic environment*.

Hypothesis 4 *Consumer ATT mediates the relationship between FSK and FSP levels in the domestic environment*.

Hypothesis 5 *Consumer BRP and ATT serially mediate the relationship between FSK and FSP levels in cross‐contamination, storage, and cooking*.

## 3 Methods

### 3.1 Sampling and Data Collection

This study collected data from two metropolitan cities (Dhaka and Chittagong) and two rural districts (Faridpur and Cox's Bazar) in Bangladesh between November 2021 and March 2022.

This study recruited consumers aged 18 years or older who prepared food at least 2–4 times per week in the domestic kitchen with a cross‐sectional descriptive survey. Monte Carlo Power Analysis for Indirect Effects (https://schoemanna.shinyapps.io/mc_power_med/) was used to estimate the minimum sample size, medium effect size, and statistical power of 80%, as suggested by Schoemann et al. (2017), and the minimum sample size was found to be 134. However, 503 data were collected as the large sample size enhances accuracy. The data collection process was underway following approval from the University Human Research Ethics Committee. Before commencing the data collection, all research assistants had extensive training according to university guidelines. The study assistants attended the locations, including local markets, bazaars, schools, colleges, and parks. A face‐to‐face survey was conducted with the participants after the researcher explained the study goals and obtained informed consent through convenience sampling.

### 3.2 Survey Instruments

The participants completed a set of questionnaires that included a sociodemographic profile and the cross‐contamination, storage, and cooking‐related knowledge (6 questions), self‐reported practice (9 questions), attitude (8 questions), and behavior‐specific risk perception (5 questions) (Evans et al. 2020; Gong et al. 2016; Levy et al. 2008; Lihan et al. 2019; Moreb et al. 2017; Odeyemi et al. 2019; Ruby et al. 2019; Soon et al. 2021, 2020; Tabrizi et al. 2017).

The participants were asked questions on their food safety knowledge (FSK), including three alternative answers: “Yes,” “No,” and “Don't know”. A correct response received one point, whereas an incorrect or “don't know” response received zero points. ‘Don't know’ was added to the multiple‐choice answers to reduce the likelihood that participants would select the correct response accidentally. The food safety practice (FSP) section includes questions employing a 5‐point Likert scale, from “never” (1 point) to “always” (5 points). The food safety attitude (ATT) includes questions on a 5‐point Likert scale, from “strongly disagree” (1 point) to “strongly agree” (5 points). The behavior risk perception (BRP) includes questions to determine the participants' perceptions of the risk of acquiring FBDs after specific behavior associated with their food safety practice on a five‐point Likert scale from “very low” to “very high”.

### 3.3 Data Analysis

The data were analyzed using SPSS software, Mac OS version 29. This study utilized descriptive statistics to summarize sociodemographic characteristics of the respondents, and frequencies, correlations, and collinearity diagnostics were also investigated before hypothesis testing.

This study used the conventional two‐step methodology (Anderson and Gerbing 1988), adapted for a regression‐based mediation framework. All latent variables (food safety knowledge, attitude, practice, and risk perception) were examined for their reliability and validity. This study used Average Variance Extracted (AVE > 0.50) for convergent validity and Composite Reliability (CR > 0.70) for construct reliability. Discriminant validity was assessed using the Fornell‐Larcker criterion, comparing AVE with Maximum Shared Variance (MSV) (Fornell and Larcker 1981), and by verifying that correlations between constructs were below 0.70.

In the second step, the hypothesized mediation effects were tested using Hayes' PROCESS macro (Hayes 2013, 2022). PROCESS Macro is a regression‐based approach that estimates direct, indirect, and total effects using bootstrapped confidence intervals. As the PROCESS macro does not estimate latent variable models or global goodness‐of‐fit indices (e.g., χ^2^/df, CFI, TLI, SRMR, and RMSEA), model adequacy was established through reliability and validity diagnostics rather than structural equation modeling fit statistics. Therefore, the adequacy of the constructs was established through reliability and validity assessments (AVE, CR, MSV, and correlations), which provided sufficient support for testing the proposed mediation pathways.

## 4 Results

### 4.1 Participant Profile

A total of 503 respondents participated in the study. Most study respondents were female (90.3%), housewives (69.4%), and aged between 30 and 39 years (39%). 48.5% and 39.6% had university and school‐level education, respectively.

In Table 1, the item‐level analysis of food safety knowledge showed notably low mean scores for several indicators, such as cross‐contamination (FSK1: *M* = 0.44), safe food handling (FSK4: *M* = 0.30), and storing (FSK6: *M* = 0.44), indicating the overall food safety knowledge among the sampled domestic food handlers was limited.

**TABLE 1:** Validity and reliability of the study constructs.

Expression	Scale item	Mean (SD)	Factor Loadings (FL)	AVE	MSV	CR	Cronbach's *α*
*Knowledge*	*FSK*			*0.633*	*0.091*	*0.90*	*0.891*
Uncovered abrasion or cuts can cause cross‐contamination of food.	FSK1	0.44 (0.497)	0.882				
It is necessary to wash the knife that has been used to cut raw meat with soap and water before using it again.	FSK2	0.85 (0.348)	0.448				
Storing raw and cooked food together can cause food contamination.	FSK3	0.47 (0.50)	0.883				
Leftover food smelling good is still safe to eat.	FSK4	0.30 (0.461)	0.835				
It is ideal not to keep leftover food in the fridge for more than 2 days.	FSK5	0.49 (0.50)	0.774				
Storing leftover food on the table or kitchen shelf is not good.	FSK6	0.44 (0.497)	0.864				
*Behavior risk perception*	*BRP*			*0.735*	*0.283*	*0.89*	*0.956*
The risk of acquiring foodborne disease is significantly increased if you do not heat food properly or eat raw food. Improper heating may not kill harmful bacteria, viruses, and parasites that can contaminate food. Similarly, raw foods, such as raw milk or eggs, can harbor these pathogens. Consuming contaminated food can lead to various foodborne illnesses, ranging from mild gastrointestinal symptoms to severe and potentially life‐threatening conditions.	BRP1	2.64 (0.846)	0.815				
What is the risk of acquiring foodborne disease if you use the same cutting board or knife to slice raw meat and to cut fruits and vegetables?	BRP2	2.66 (0.869)	0.885				
What is the risk of acquiring foodborne disease if you keep food in the room temperature for more than 2 h?	BRP3	2.75 (0.865)	0.871				
*Practice*	*FSP*			*0.576*	*0.249*	*0.84*	*0.793*
Do you use different cutting boards or knife to slice raw meat and to cut fruits and vegetables?	FSP1	1.76 (0.987)	0.565				
Do you use different towel to wipe kitchen surfaces and to dry your hands?	FSP2	3.98 (1.653)	0.696				
Do you prefer keeping the meal at refrigerator, if your family member is going to be several hours late for a hot meal?	FSP3	3.15 (1.21)	0.879				
Do you reheat leftover food until it is boiling hot?	FSP4	3.50 (0.995)	0.855				
*Attitude*	*ATT*			*0.671*	*0.283*	*0.93*	*0.919*
Wearing accessories like rings, bracelets are not fine when cooking food.	ATT1	2.92 (1.267)	0.725				
It is necessary to cover your cut with bandage and use gloves while preparing food.	ATT2	4.20 (0.766)	0.887				
It is unhealthy to taste dish out food with unprotected hands.	ATT3	3.8 (1.079)	0.849				
Use cutting board and knives set for meat and another set for vegetables.	ATT4	3.69 (1.138)	0.843				
It is necessary to read conditions of use and storage of packaged food.	ATT5	4.36 (0.703)	0.881				
I don't prefer half‐boiled or half‐cooked eggs.	ATT6	3.45 (1.25)	0.742				
It is unhealthy to consume expired food.	ATT7	4.50 (0.696)	0.793				

*Note:* Discriminant validity is established when AVE > MSV (Fornell and Larcker 1981).

Abbreviations: AVE, average variance extracted; CR, composite reliability; MSV, maximum shared variance.

### 4.2 Measurement Model

To assess the items' competency with variance, the Kaiser‐Meyer‐Olkin (KMO) and Bartlett's tests of sphericity were run. The KMO test is employed to check sample adequacy and applicability for factor analysis to demonstrate the level of variance among the variables. The correlation matrix is compared to the identity matrix using Bartlett's test of sphericity to determine whether there is a significant difference. The test findings were 0.862 (> 0.60) and significant (*p* < 0.001), suggesting the presence of some correlations between the variables and the lack of an identity correlation matrix, respectively. This study conducted exploratory factor analysis (principal component with varimax rotation) and internal consistency tests to verify the convergent and discriminant validity of the study measures. At the 0.4 cut‐offs, all factor loadings were significant (Hair et al. 2010). Accordingly, two items from behavior risk perception, five from self‐reported practice, and one from attitude were removed.

Scale items, exploratory factor analysis findings, and internal consistency data are included in Table 1. Each factor is based on its underlying construct. Internal consistency reliability was evaluated using Composite Reliability (CR) and Cronbach's alpha (*α*). Table 1 results indicated good reliability [CR > 0.70 (Cheung et al. 2023); *α* > 0.70 (Taber 2018)] in all measures. The validity of the constructs was confirmed by the average variance extracts (AVE > 0.5) (Rothstein et al. 1990). Table 1 shows that convergent validity was supported, as the AVE values for food safety knowledge (AVE = 0.633), attitude (AVE = 0.671), practice (AVE = 0.576), and behavioral risk perception (AVE = 0.735) were all above the recommended minimum of 0.50.

Discriminant validity was assessed using two criteria. First, inter‐construct correlations were examined. The correlations of the study variables indicate that food safety knowledge, practice, behavior risk perception, and education are all significantly correlated, except for the relationship between gender and age. Food safety knowledge (FSK) was significantly positively correlated with behavior risk perception (BRP) (*r* = 0.302, *p* < 0.01), food safety practice (FSP) (*r* = 0.259, *p* < 0.01), and food safety attitude (ATT) (*r* = 0.119, *p* < 0.05). Similarly, BRP was positively correlated with FSP (*r* = 0.499, *p* < 0.01) and ATT (*r* = 0.532, *p* < 0.01). As the correlations between the research variables are lower than 0.70, the findings of the correlation analysis offer additional support for the claim that discriminant validity exists. Second, discriminant validity was assessed by comparing the Average Variance Extracted (AVE) of each construct with its Maximum Shared Variance (MSV). As shown in Table 1, the AVE values for FSK, ATT, FSP, and BRP were all greater than their corresponding MSVs (0.091, 0.283, 0.249, and 0.283, respectively) (Fornell and Larcker 1981; Hair et al. 2010). Using the variance inflation factor (VIF < 3.5), collinearity was confirmed (Rothstein et al. 1990). All VIF values for the predictor are less than 5, which alleviates the multicollinearity issue in the regression model. The findings suggest that the measurement model is reliable and demonstrates both convergent and discriminant validity. These support using these constructs in the subsequent mediation analysis with the PROCESS Macro.

### 4.3 Regression Analysis

Hayes (2022) PROCESS macro was applied to evaluate the association between food safety knowledge (FSK), attitude (ATT), practice (FSP), and behavior risk perception (BRP). The mediator variable is considered to have a mediating impact if Haye's (Hayes 2013) Macro Process bootstrapping approach identifies the indirect effect (IE) of the independent variable (*X*) on the dependent variable (*Y*) through the mediator (*M*) and the bias‐corrected 95% CI around the IE from 5000 bootstrap resamples. If the bias‐corrected 95% confidence interval for the indirect effect (IE) excludes zero, it is deemed statistically significant.

**FIGURE 2:**
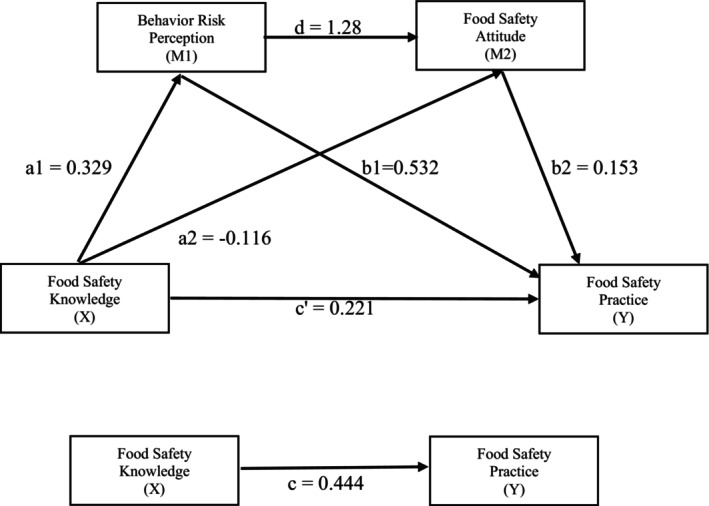
PROCESS serial Mediation model. a1 = effect of *X* on M1; b1 = effect of M1 on *Y*; a2 = effect of *X* on M2; b2 = effect of M2 on *Y*; *d* = effect of M1 on M2; *c* = total effect of *X* on *Y*; *c*' = direct effect of *X* on *Y*, controlling for mediators.

In Figure 2, path a1 indicates the direct relationship that FSK exerts a significant positive effect on BRP (*β* = 0.329, *p* < 0.001), supporting Hypothesis 1. Path b1 shows that BRP was significantly and positively associated with FSP (*β* = 0.532, *p* < 0.001). Simultaneously, the total effect of FSK on FSP was significant in path *c* (*β* = 0.444, 95% CI: 0.298 to 0.589). The direct effect of FSK on FSP (path *c*') remained significant after controlling for the mediator (effect = 0.221, *p* < 0.001, 95% CI: 0.089 to 0.354; Tables 2 and 3). Bootstrap mediation analysis indicated that the indirect effect of FSK on FSP through BRP was statistically significant (indirect effect = 0.175, 95% CI: 0.114 to 0.243; Table 3), as the confidence interval did not include zero. Hence, BRP is considered a mediator for FSK on FSP, supporting Hypothesis 3. In contrast, the direct path a2 shows that the association between FSK and ATT was not significant (*β* = −0.116, *p* = 0.251) (Table 2) (Figure 2). Thus, rejecting Hypotheses 2 and 4. Finally, BRP was significantly positively associated with ATT (*β* = 1.28, *p* < 0.001) and FSP (*β* = 0.532, *p* < 0.001), establishing a serial mediation pathway supporting Hypothesis 5 (Figure 2).

**TABLE 2:** The process macro‐mediation model shows the regression analysis of variable relationships.

Outcome variable	Predictor variable	*β*	SE	*t*	*p* (CI)	
BRP	FSK	0.329	0.046	7.09	< 0.001*** (0.238, 0.420)	*R* ^2^ = 0.091 *F* = 50.34 *p* < 0.001
ATT	FSK	−0.116	0.101	−1.14	0.251 (−0.316, 0.083)	*R* ^2^ = 0.285 *F* = 99.72 *p* < 0.001
	BRP	1.28	0.093	13.76	< 0.001*** (1.10,1.46)	
FSP	FSK	0.221	0.067	3.28	0.001** (0.089, 0.354)	*R* ^2^ = 0.299 *F* = 71.03 *p* < 0.001
	BRP	0.532	0.072	7.31	< 0.001*** (0.389, 0.675)	
	ATT	0.153	0.029	5.18	< 0.001*** (0.095, 0.212)	

Abbreviations: ATT, food safety attitude; BRP, behavior risk perception; CI, 95% confidence interval; FSK, food safety knowledge; FSP, food safety practice.

** *p* < 0.01.

*** *p* < 0.001.

**TABLE 3:** The PROCESS mediation model shows the total, direct, and indirect effect of food safety knowledge (FSK) on food safety practice (FSP).

	Effect (*β*)	Boot SE	Boot LLCI	Boot ULCI
Total effect	0.444	0.074	0.298	0.589
Direct effect	0.221	0.067	0.089	0.354
Total indirect effect	0.222	0.038	0.149	0.298
*Indirect effect 1*				
FSK → BRP → FSP	0.175	0.033	0.114	0.243
*Indirect effect 2*				
FSK → ATT → FSP	‐ 0.018	0.016	‐ 0.050	0.013
*Indirect effect 3*				
FSK → BRP → ATT → FSP	0.065	0.016	0.037	0.099

*Note:* Boot SE, standard error; Boot LLCI, lower bounds; Boot ULCI, upper bounds of the 95% confidence intervals indirect effects estimated by the bootstrap method. Unstandardized effects are reported. Bootstrapped confidence intervals are based on 5000 resamples.

In Table 3, the bootstrap test results showed that BRP mediated the relationship between FSK and FSP, with a total indirect effect of 0.222 and a 95% CI (0.149–0.298) that excluded zero. Specifically, the serial mediating effect was composed of an indirect effect generated by FSK → BRP → ATT → FSP (effect = 0.065, 95% CI: 0.037–0.099). In addition, the direct effect of FSK on FSP remained significant after including the mediators (effect = 0.221, 95% CI: 0.089–0.354), indicating partial mediation as both the total and indirect effects were significant (Table 2). Hence, it can be concluded that food handlers' behavior risk perception of food safety partially mediated the relationship between their food safety knowledge and practice, and there is partial serial mediation of behavior risk perception and attitude on the relationship between knowledge and practices. This total indirect effect represents approximately 50% of the total effect, calculated by dividing the total indirect effect (0.222) by the total effect (0.444), indicating that about half of the influence of FSK on FSP operates via BRP and ATT.

## 5 Discussion

This study aims to evaluate how consumer cross‐contamination, storage, and cooking‐related food safety knowledge in the domestic environment is associated with their behavior risk perception, and attitudes toward food safety and food safety practices. An extended KAP model was proposed, with the construct risk perception to understand consumer behavior.

Confirming Hypothesis 1, the present research identified that consumer food safety knowledge is positively associated with their perceived risk of food safety behavior. The proposed model has evaluated a positive impact on the knowledge‐risk perception relationship. Food safety knowledge showed a significant positive effect on behavior risk perception (*β* = 0.329), whereas its effect on attitude (ATT) was not statistically significant (*β* = −0.116). Thus, it can be concluded that the awareness of food safety is enhanced when consumer knowledge of food handling interferes with perceived risk. This finding aligns with earlier studies that stated knowledge is a prerequisite for assessing the risk factors (De Boer et al. 2005). Several researchers also mentioned knowledge as a crucial factor that affects risk evaluation (Fife‐Schaw and Rowe 1996; Frewer et al. 1994; McCarthy et al. 2007).

Studies have highlighted that knowledge is the factor that regulates appropriate food safety practices (Lim et al. 2016; Mucinhato et al. 2022). Several elements, including knowledge (Gong et al. 2016), attitude (Ajzen et al. 2007), and risk perception (Young et al. 2017), impact a reasoned choice process that leads to a correct food safety practice (Smith et al. 2007). This study found that consumer knowledge of food safety has a direct and positive association with their food safety practices. This finding is similar to Lim et al. (2016), who discovered that knowledge directly impacts behavior. Although knowledge is imperative, this is inadequate for changing behavior (Taché and Carpentier 2014). In addition to knowledge, the perception of risk motivates consumers to act, including avoidance, prevention, adaptation, or even neglect of the associated risks (Wachinger et al. 2013). Thus, risk perception guides practices (McCarthy et al. 2007; Parra et al. 2014), supporting this study finding that revealed consumer perceived behavior risk of food safety is positively related to their food safety practices (Table 4).

**TABLE 4:** Results of hypothesis testing.

Hypotheses	Hypotheses path	Decision
Hypothesis 1	FSK → BRP	Supported
Hypothesis 2	FSK → ATT	Not supported
Hypothesis 3	FSK → BRP → FSP	Supported
Hypothesis 4	FSK → ATT → FSP	Not supported
Hypothesis 5	FSK → BRP → ATT → FSP	Supported

Abbreviations: ATT, food safety attitude; BRP, behavior risk perception; FSK, food safety knowledge; FSP, food safety practice.

The current mediation analysis revealed that consumer behavior‐specific risk perception partially mediated the relationship between food safety knowledge and practice, supporting Hypothesis 3. As the consumer knowledge in this study directly and indirectly (via behavior risk perception) acts on food safety practices, behavior modifications occurred in both processes from the direct effect of knowledge and through the mediator variable behavior risk perception. Studies suggest that enhancing communication to increase risk perception and modify general behavior is the optimal strategy for improving practices (Byrd‐Bredbenner et al. 2013; McCarthy et al. 2007; Parra et al. 2014) (Table 4).

On the other hand, this study explored that consumer cross‐contamination, storage, and cooking‐related knowledge and attitude toward food safety were not statistically significant (Hypothesis 2). Similar findings were found by Rebouças et al. (2017) and Akabanda et al. (2017), showing that improved food safety knowledge has not led to a rise in positive attitudes toward food safety. Contrarily, Taha et al. (2020) found a significant positive relation between food safety knowledge and attitude. This study also found no evidence of a mediating role for food handler attitudes in the relationship between their knowledge of food safety and practice (Hypothesis 4). This result contrasts with Ko's (2013) study, which found that positive attitudes are essential for food handlers' conversion of food safety knowledge into proper food safety practices, mediating between food safety knowledge and practices.

A particularly important contribution of this study is the lack of support for Hypothesis 2 (FSK → ATT) and Hypothesis 4 (FSK → ATT → FSP), which challenges the core assumption of the traditional Knowledge–Attitude–Practice (KAP) model. Although the KAP framework assumes that increased knowledge naturally leads to favorable attitudes and, subsequently, safer practices (Mihalache et al. 2021; Zanin et al. 2017), the present findings demonstrate that food safety knowledge alone did not significantly influence consumers' attitudes toward food safety, nor did attitude mediate the relationship between knowledge and practice. This non‐finding is critical, as it highlights a persistent gap between what consumers know and how they feel and behave in domestic food handling contexts.

Importantly, this study observed notably low mean scores for several food safety knowledge items, which represent a critical finding and warrant explicit discussion. Many domestic food handlers demonstrate limited knowledge of basic food safety issues, particularly regarding cross‐contamination and safe handling practices, despite being the primary food handlers. These knowledge insufficiencies are concerning, as they are the most common behaviors for transmitting foodborne diseases in domestic settings. The observed low knowledge levels might explain why food safety knowledge alone showed a weaker direct association with attitudes and practices, reinforcing the argument that knowledge acquisition, in isolation, is insufficient to produce meaningful attitudinal change or behavioral compliance.

Together, these findings underscore a key limitation of the traditional KAP model and provide strong empirical justification for extending the framework. The results highlight the importance of incorporating behavior‐specific risk perception as a more proximal and influential mechanism through which food safety knowledge translates into safer practices. Consequently, this study emphasizes the urgent need for targeted, practical food safety education and risk communication interventions that focus on high‐risk domestic practices rather than just raising general awareness.

Confirming Hypothesis 5, the main result of this study was that the behavior risk perception (BRP) and attitude (ATT) toward food safety partially mediated the relationships between food safety knowledge and practice through a serial mediation pathway. The BRP → ATT path showed a significantly robust effect (*β* = 1.28) compared to the original KAP (FSK → ATT → FSP) model. Risk perception is the basis of an individual's attitudes, and risk judgments include what individuals believe and feel about this risk. When food handlers perceive risky behavior related to food handling practices, a positive attitude toward food safety practices develops. Thus, increased risk perception toward incorrect food handling behavior influences positive food safety attitudes and, eventually, the food safety practice in the kitchen (van der Vossen‐Wijmenga et al. 2022). This study applies to the BRP ➝ ATT sequence, although risk perception and attitude may have a reciprocal effect. Food safety attitudes are shaped by immediate cognitive and affective evaluations that are more likely to be triggered by behavior‐specific risk perception associated with specific food handling behaviors. Furthermore, the model's strong and significant BRP → ATT path validates the notion that risk perception is a more proximal antecedent of attitude in the case of domestic food safety. A few previous studies have examined the role of perceived risk and attitude in the relationship between knowledge and practice toward food safety; however, they have not adequately investigated the mechanism of this serial mediation path. An extended TPB model by Mucinhato et al. (2022) revealed that perceived risk is strongly related to food safety attitudes in domestic food safety practices during the COVID‐19 pandemic. Further, considering the influence of attitudes on practices, this study shows that these constructs are significantly and positively related, aligning with previous research (Al‐Kandari et al. 2019; Aquino et al. 2021; Asmawi et al. 2018). This serial mediation model in this study contributes novel perspectives on the consequences of consumer cross‐contamination, storage, and cooking‐related food safety knowledge, with their perceived behavior‐specific risk and attitude toward food safety mediating the effect of knowledge on practice.

## 6 Implications

Based on the expanded KAP model, this study attempts to comprehend safe food handling procedures. This study extends the knowledge‐risk perception‐attitude‐practice paradigm to household food handlers in Bangladesh. The study model included behavior risk perception of adult consumers concerning food safety in the KAP model. The main difference is that this study offers an enhanced comprehension of food safety procedures and presents a range of significant elements (e.g., knowledge, behavior risk perception, and attitude) for creating effective health education initiatives mitigating the hazards linked to inappropriate food handling in the household. The significance of the behavior risk perception shows the importance of food handling errors influencing food safety practices. The findings imply that raising awareness of the risk associated with specific unsafe food handling practices may encourage consumers to take more precautions in their food safety behavior. This study tested the regression coefficient and significance of the knowledge‐risk perception‐attitude‐practice model using the PROCESS macro model for serial mediation analysis, contrasting earlier studies that predicted safe food handling practices based on two constructs: knowledge and attitude. The findings of this study indicate that the model performs well compared to the original KAP model in the Bangladeshi context, demonstrating the model's applicability globally for better food handling practices. This study adds to the existing body of literature on food safety by illustrating the beneficial influence of risk perceptions and attitudes on household food handling practices.

## 7 Study Limitations and Future Research

The cross‐sectional nature of the study is an evident constraint of this study. Due to their non‐randomized nature and the indeterminate directionality between associated variables, cross‐sectional studies cannot determine causality conclusively. Although a large sample size improves statistical power and the precision of the estimates, it does not surpass the limitation of causal inference in the cross‐sectional design. Nonresponse and recall bias were mitigated by collecting data from trained research assistants and allowing sufficient time for respondents to recall (Ishra et al. 2025b). The inference of causal relationships between the variables of the study could be impacted due to the use of self‐reported data in the analysis. When conducting surveys on behavior or practice in a large population, self‐reported data are more feasible than observation. Future research can evaluate food handling behaviors more precisely by using observational approaches. The sampling composition represents yet another significant constraint. The results' generalizability is limited by using a sample that is significantly biased toward women (90.3%) and housewives (69.4%). Nonetheless, the results offer important insights into domestic food handling behaviors in Bangladesh, where women are primarily responsible for food preparation in households. However, rather than representing all adult consumers, the results mostly represent the attitudes and behaviors of female domestic food handlers. Future studies may employ probability‐based sampling methods and include more diverse populations, particularly male food handlers and individuals from varied socioeconomic and occupational backgrounds, to strengthen external validity and broaden the applicability of the findings. Another limitation was the inability to generalize the findings to food handlers in Bangladesh due to the use of a convenience sample approach. The scope of this empirical analysis restricts the behavior risk perceptions to a single psychometric variable. Future researchers should expand upon previous data collection efforts by measuring risk perceptions as a multidimensional concept and observing the incongruity between self‐reported behaviors and factual observations. Exploring the effectiveness of behavior‐specific risk communication strategies and identifying behavior‐specific food safety risks should be addressed in this community. Future studies may aim to enhance this serial mediation effect of food safety risk perception and attitude by employing a risk perception attitude framework (RPA) for a better understanding of the effect. The least effective components that contributed to the consumer's intent to practice food safety were attitude and food safety knowledge. It is advised that future research should focus on moderators that can enhance these aspects, such as age, gender, and experience.

## 8 Conclusion

This study provides empirical evidence that challenges the linear assumptions of the traditional Knowledge–Attitude–Practice (KAP) model in the context of domestic food safety. Food safety knowledge by itself does not consistently convert into positive attitudes or safer food handling behaviors, as seen by the absence of support for the direct knowledge–attitude pathway (Hypothesis 2) and the attitude‐mediated pathway between knowledge and practice (Hypothesis 4). This research reveals a crucial gap between acquiring new knowledge and changing one's behavior, especially in everyday household routines that are influenced by context and habit. The extended model, on the other hand, demonstrates that behavior‐specific risk perception has a more direct and significant impact on the relationship between knowledge and practice, both directly and through attitudes (Hypotheses 3 and 5). This study underscores the necessity of adopting risk‐based, behavior‐specific communication techniques alongside the implementation of knowledge‐focused interventions to effectively improve food safety practices in domestic settings.

## Author Contributions

**Rakia Ishra:** conceptualization, methodology, data collection and curation, formal analysis, writing – original draft, and visualization. **Saif Sharif:** data collection and curation, formal analysis, and writing – review and editing. **Jeffrey Soar:** supervision, validation, and writing – review and editing. **Rasheda Khanam:** supervision, validation, and writing – review and editing.

## Funding

The authors have nothing to report.

## Ethics Statement

Ethical approval was obtained from the Australian University of Southern Queensland Human Research Ethics Committee (USQ HREC ID:H21REA161).

## Consent

Appropriate Informed consent was taken from the participants.

## Conflicts of Interest

The authors declare no conflicts of interest.

## Data Availability

The data supporting this study's findings are available from the corresponding author upon reasonable request.
